# Increased Soluble Interleukin 6 Receptors in Fabry Disease

**DOI:** 10.3390/jcm13010218

**Published:** 2023-12-29

**Authors:** Livia Lenzini, Elisabetta Iori, Monica Vettore, Giorgia Gugelmo, Claudia Radu, Andrea Padoan, Gianni Carraro, Paolo Simioni, Lorenzo Calò, Angelo Avogaro, Gian Paolo Rossi, Nicola Vitturi

**Affiliations:** 1 Internal & Emergency Medicine Unit, Department of Medicine, Padova University Hospital, 35128 Padova, Italy; livia.lenzini@unipd.it (L.L.); gianpaolo.rossi@unipd.it (G.P.R.); 2 Division of Metabolic Diseases, Department of Medicine, Padova University Hospital, 35128 Padova, Italy; elisabetta.iori@unipd.it (E.I.); vettore.monica@unipd.it (M.V.); angelo.avogaro@unipd.it (A.A.); 3 Division of Clinical Nutrition, Department of Medicine, Padova University Hospital, 35128 Padova, Italy; giorgia.gugelmo@unipd.it; 4 General Medicine and Thrombotic and Hemorrhagic Diseases Unit, Department of Medicine, Padova University Hospital, 35128 Padova, Italy; claudiamaria.radu@unipd.it (C.R.); paolo.simioni@unipd.it (P.S.); 5 Laboratory Medicine Unit, Department of Medicine, Padova University Hospital, 35128 Padova, Italy; andrea.padoan@unipd.it; 6 Nephrology, Dialysis and Transplant Unit, Department of Medicine, Padova University Hospital, 35128 Padova, Italy; giannicarraro63@gmail.com (G.C.); renzcalo@unipd.it (L.C.)

**Keywords:** Fabry disease, interleukin 6 receptor, exosomes, inflammation

## Abstract

Fabry disease (FD) is an X-linked lysosome storage disease that results in the accumulation of globotriaosylceramide (Gb3) throughout the body leading to irreversible target organ damage. As the role of secondary mediators (inflammatory molecules) and their mechanisms has not been fully elucidated, we focused on the interleukin (IL)-6 system in adult FD patients and in matched healthy subjects. To obtain insights into the complex regulation of IL-6 actions, we used a novel approach that integrates information from plasma and exosomes of FD patients (n = 20) and of healthy controls (n = 15). Soluble IL-6 receptor (sIL-6R) levels were measured in plasma with the ELISA method, and membrane-bound IL-6R was quantified in plasma and urinary exosomes using flow cytometry. In FD patients, the levels of soluble IL-6R in plasma were higher than in control subjects (28.0 ± 5.4 ng/mL vs. 18.9 ± 5.4 ng/mL, *p* < 0.0001); they were also higher in FD subjects with the classical form as compared to those with the late-onset form of the disease (36.0 ± 11.4 ng/mL vs. 26.1 ± 4.5 ng/mL, *p* < 0.0001). The percentage of urinary exosomes positive for IL-6R was slightly lower in FD (97 ± 1 vs. 100 ± 0% of events positive for IL-6R, *p* < 0.05); plasma IL-6 levels were not increased. These results suggest a potential role of IL-6 in triggering the inflammatory response in FD. As in FD patients only the levels of sIL-6Rs are consistently higher than in healthy controls, the IL-6 pathogenic signal seems to prevail over the homeostatic one, suggesting a potential mechanism causing multi-systemic damage in FD.

## 1. Introduction

Fabry disease (FD) (OMIM: 301500) is an X-linked lysosome storage disease due to deficient or absent activity of the lysosomal enzyme alpha-galactosidase A (GLA). This genetic defect causes the accumulation of globotriaosylceramide (Gb3) and related glycosphingolipids in the plasma and lysosomes of the vessels, nerves, tissues, and organs throughout the body [1]. The disease can present with two phenotypes, classic and late-onset FD, differing in the percentage of the residual GLA activity. The latter is almost absent (<3%) in the classic form, while it is up to 25% in the late form. These differences impact the rate of Gb3 accumulation in cells, which is higher when the GLA residual activity is almost absent (classic form) and, clinically, translates into earlier onset of clinical signs and differences in the severity of the damage to target organs. The rate of Gb3 accumulation in the lysosomes influences the toxic effects on cells and promotes different degrees of inflammatory response and fibrotic processes, which translate into hypertrophic cardiomyopathy, chronic proteinuric nephropathy, recurrent strokes, and premature death in the most severe presentations. Moreover, in heterozygous females, GLA activity is often normal because of the random X-inactivation and the symptoms can be mild, further increasing the challenges for the diagnosis and treatment of FD [2]. As regards the currently available specific and targeted treatments, which are enzyme replacement therapy (ERT) and chaperone therapy in patients with amenable mutations, early intervention is crucial because disease progression is common in patients with FD and reversal of established organ damage is hardly feasible [2].

Besides the toxic role of Gb3 and related glycosphingolipids accumulation in cellular damage, inflammatory and oxidative molecules [3,4,5,6] have been pointed out as key secondary mediators in the mechanisms leading to irreversible damage in multiple organ systems in FD [7]. High levels of proinflammatory molecules such as TNF alpha and IL-6 have been reported in FD patients [8,9,10,11,12].

IL-6 is a pleiotropic cytokine involved in several physiological and pathological conditions. Its action is mediated by two central signaling systems: classical IL-6 signaling acts through the binding of IL-6 to the membrane-bound IL-6 receptor (IL-6R) α-subunit and the glycoprotein 130 (gp130) signal-transducing subunit. At the same time, IL-6 trans-signaling is activated in the presence of complexes of IL-6 and the soluble form of the IL-6 receptor (sIL-6R) linked to the membrane-bound gp130 [13].

The activation of these two signaling systems have different effects on target cells. The binding of IL-6 to membrane receptor IL-6R, expressed by hepatocytes, muscle, immune, and epithelial cells, triggers mechanisms involved in homeostasis and in acute inflammatory conditions, while IL-6 trans-signaling has emerged as the predominant pathway by which IL-6 promotes disease [14].

The release of sIL-6Rs into the blood flow can occur via different molecular mechanisms, including protein synthesis after alternative mRNA splicing [15] and cleavage of the transmembrane domain of the IL-6R by proteases, such as ADAM10 and ADAM17 [16,17].

Isolation of microvesicles, i.e., exosomes, from human serum revealed that they carry the full-length IL-6R. Hence, even cells that lack IL-6R expression can become responsive to IL-6 via fusion of extracellular vesicles presenting IL-6R on their surface [18]. To obtain insights into the complex equilibrium of the soluble or membrane-bound forms of IL-6 receptors, exosomes, which derive from the secretion of membranes and enclosed intracellular parts of parental cells, represent a unique tool for ex vivo measurement of the levels of membrane-bound IL-6Rs.

Thus, this study was aimed at investigating if the levels of IL-6 and of its receptors were altered in FD by integrating information deriving from plasma and exosomes collected from adult patients with FD and from matched healthy subjects.

## 2. Materials and Methods

### 2.1. Collection of Blood and Urine Samples

Adult patients with a biochemical and a genetic diagnosis of Fabry disease were referred to the Adult Metabolic Diseases Center of the University Hospital of Padova. Blood and urine from patients and healthy subjects were processed and stored according to our local Institutional Ethical Committee procedures (Approval date 11 December 2006). All subjects provided written informed consent before participation.

Specimens were collected into test tubes without or with anticoagulant (EDTA) and centrifuged at 2500 rpm for 15 min at +4 °C. The supernatant (serum or plasma) was transferred to clean test tubes and stored at −80 °C before being assayed.

### 2.2. Isolation and Characterization of Exosomes from Urine and Blood

Exosomes from the 40–50 mL of urine and 4–6 mL of plasma from healthy adult subjects and adult FD patients were isolated with ultracentrifugation [19].

Briefly, after the first centrifugation at 1000× *g* for 10 min at 4 °C, urine supernatant was ultra centrifuged (OPTIMA Max XP, Beckman Coulter, Milan, Italy) for 2 h at 200,000× *g* at 4 °C. Then, after removing the supernatant, the pellet was washed with filtered PBS and ultra centrifuged for 2 h at 200,000× *g* at 4 °C and suspended in 200 µL PBS with 20 µL protease inhibitor (Cod. AMab271306, Prodotti Gianni Srl, Milan, Italy) and stored at −80 °C for further analyses.

As regards plasma, after the first centrifugation at 400× *g* for 20 min at 4 °C, supernatant was ultra centrifuged (OPTIMA Max XP, Beckman Coulter, Milan, Italy) for 20 min at 5000× *g* at 4 °C and for 90 min at 120,000× *g* at 4 °C. Then, after removing the supernatant, the pellet was washed with filtered PBS and suspended in 200 µL PBS with 20 µL protease inhibitor (Cod. AMab271306, Prodotti Gianni Srl, Milan, Italy) and stored at −80 °C for further analyses.

Transmission electron microscopy (TEM) was used to provide morphologic information on exosomes. Standard TEM protocols were used on a FEI Tecnai G2 microscope at the facility of the Department of Biology, University of Padova, Padova, Italy.

We tested the integrity of the urinary exosomes and their recovery after the centrifugation phases, incubating them with 20 μM of calcein-AM (referred to calcein-green, cod. 206700, Sigma-Aldrich, St. Louis, MI, USA) at 37 °C for 20 min. Flow cytometric analysis was performed using the CytoFLEX S flow cytometer (Beckman Coulter, Pasadena, CA, USA). Flow cytometry calibration for exosomes detection was performed with fluorescent polystyrene beads: Gigamix, a mix 1:1 of Megamix FSC and SSC Plus of known sizes of 0.1, 0.16, 0.2, 0.24, 0.3, 0.5, and 0.9 µm (BioCytex, Marseille, France) through the “Violet side Scatter” (VSSC) and fluorescent FL1 channels. VSSC at 405 nm is used to discriminate background noise. The working gate was set between 50 and 150 nm, defined as the Exosome gate.

### 2.3. Interleukin 6 and IL-6 Receptors Quantification in Blood and in Exosomes

IL-6 was measured with a chemiluminescent assay (CLIA) using the Maglumi 2000 plus (Snibe Diagnostics, Shenzhen, China) fully automated system. As declared by the manufacturer, the assay Limit of Blank was 0.5 pg/mL, while IL-6 values below 7 pg/mL were considered negative.

The soluble form of IL-6R (sIL-6R) was quantified in plasma using an ELISA kit (DEIA5605 Creative Diagnostic, D.B.A. Italia s.r.l., Segrate, Milan, Italy) specific for the plasma form, following the manufacturer’s protocol. The assay was performed using 100 µl of diluted plasma. Samples were analyzed on a plate reader (Mithras LB940 Berthold, Milan, Italy) acquiring at 450 nm.

Membrane-bound IL-6R was quantified in exosomes via flow cytometric analysis. The following antibodies for exosomes analysis were used: mouse monoclonal CD-81, PE-conjugated (#349506, BioLegend Prodotti Gianni Srl), mouse monoclonal CD-63, FITC-conjugated (# 353006, BioLegend, Prodotti Gianni Srl), and rabbit monoclonal IL-6 receptor (ab222101, Abcam, Prodotti Gianni Srl). The primary antibody against IL- 6R was detected via incubation with anti-rabbit IgG Alexa Fluor 647 conjugated antibodies (Thermo Fisher Scientific). Panels are constructed with 20 µL of exosomes in PBS, to which the following was added: 2 µL of anti-CD-81, anti-CD-63 antibodies, and anti-IL-6R. The antibodies were incubated for 30 minin the dark at room temperature (RT). Samples were analyzed with CytoFLEX after adding 100 µL of filtered PBS. The working gate was set between 50 and 150 nm, defined as the Exosome gate.

Exosomes were expressed as positive events/µL. Files were analyzed with CytExpert (Software Version 1.2, Beckman Coulter). The quantification of IL-6R was performed by calculating the percentage of positive IL-6R events compared to the CD63 positive events.

The same samples were incubated with the specific isotype-matched control antibodies (mouse and rabbit) to exclude non-specific staining. Moreover, samples were treated with 0.5% Triton X-100 (Sigma-Aldrich), a lipid solubilizing detergent, for 10 min at RT and compared with the nontreated samples to confirm that exosomes detected with flow cytometry were lipid membrane vesicles. Prior to staining, the antibodies were centrifuged at 20,000× *g* for 30 min to remove fluorescent particles. The buffers used were sterilized through a 0.2 μm filter to reduce background noise.

### 2.4. Statistical Analysis

The values obtained are reported as the mean ± Standard Deviation (SD) of at least 3 technical replicates. Statistical differences were tested with *t*-tests and ANOVA tests using GraphPad Prism ver 9.4.0. *p* < 0.05 was considered significant.

## 3. Results

### 3.1. Demographic and Clinical Data of FD Patients

In this study, we recruited 20 (12 females and 8 males) adult FD patients and 15 healthy adult subjects (8 females and 7 males). Volunteers were age and gender matched. No included subjects had signs of active inflammatory diseases or history of chronic inflammatory diseases (i.e., rheumatologic disease) at the time of blood and urine collection. FD patients presented with different *GLA* mutations (Table 1) and 70% of them had a late-onset form of the disease. A total of 70% were treated: 50% were in enzyme replacement and 20% were in chaperonic therapy.

### 3.2. IL-6 and IL-6R Levels in FD and Healthy Subjects

In FD patients, IL-6 levels were not increased compared to healthy controls; in all samples, the values were below 7 pg/mL.

Before choosing the best source of exosomes as systemic indicators of the membrane-bound IL-6R, we preliminarily compared the levels of membrane-bound IL-6R in exosomes collected from the blood and urine of a subset of FD patients (n = 9). Based on the flow cytometry analysis, no difference was found in the percentage of events that were positive for membrane-bound IL-6R between the blood- and urine-derived exosomes: 96 ± 1 vs. 97 ± 1% of events positive for IL-6R, *p* = 0.515, respectively. Thus, we performed the next analyses on urinary exosomes. In FD subjects (n = 17), the percentage of urinary exosomes positive for IL-6R was slightly lower than in control subjects (n = 15): 96 ± 1 vs. 100 ± 0% of events positive for IL-6R, *p* < 0.05) (Supplementary Materials).

We did not find any significant relation among IL-6, membrane IL-6R levels, *GLA* mutation status, age, gender, FD specific (ERT or chaperonic) and other therapies, kidney disease biomarkers, levels of lyso-Gb3, and the presence of a classical or a late form of FD.

**Figure 1:**
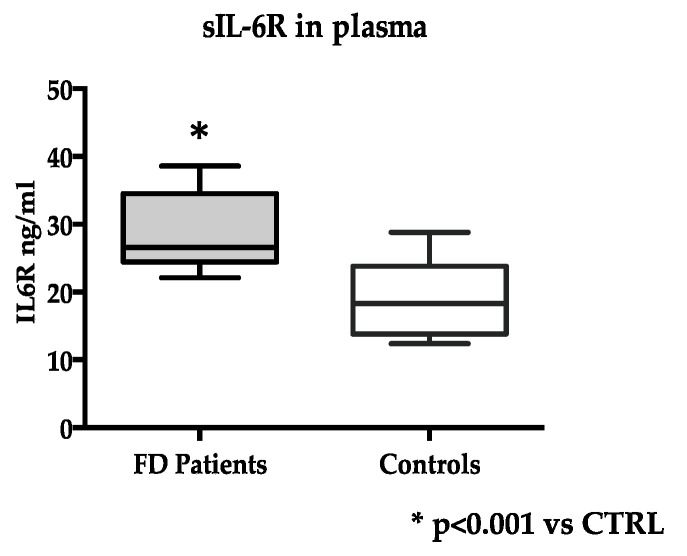
Soluble IL-6R (sIL-6R) levels in FD (n = 20) and control subjects (n = 15). Measurements were performed in duplicate. Data are reported as mean ± s.d.

At variance, the levels of soluble IL-6R in plasma were significantly higher in FD (n = 20) than in control subjects (n = 15): 28.0 ± 5.4 ng/mL vs. 18.9 ± 5.4 ng/mL, *p* < 0.0001 (Figure 1).

### 3.3. Soluble IL-6R Levels and FD Patients’ Characteristics

**Figure 2:**
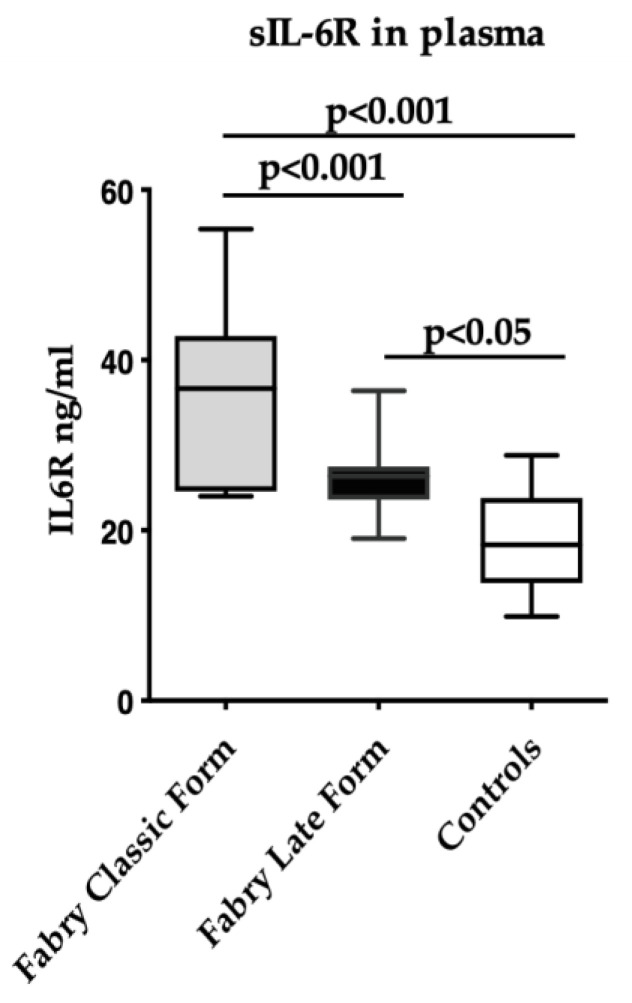
Soluble IL-6R levels in FD with the classical form (n = 6), with late-onset form (n = 14), and in control (n = 15) subjects. Measurements were performed in duplicate. Data are reported as mean ± s.d.

Soluble IL-6R levels were higher in FD subjects with the classical form (n = 6) as compared to FD with the late-onset form (n = 14) (36.0 ± 11.4 ng/mL vs. 26.1 ± 4.5 ng/mL, *p* < 0.0001) (Figure 2).

No difference was found in the levels of soluble IL-6R between FD patients with (n = 14) and those without (n = 6) therapy (29.5 ± 9.6 ng/mL vs. 30.0 ± 5.9 ng/mL, *p* < 0.0001).

We performed a retrospective subgroup analysis in eight FD patients, measuring soluble IL-6R before starting ERT and after 3 years of treatment. We did not detect any significant difference in soluble IL-6R between the groups (27.57 ± 5.64 ng/mL vs. 25.97 ± 4.60 ng/mL, *p* = 0.545).

Finally, no other relation was found amongst soluble IL-6R with the *GLA* mutation status, age, gender, FD specific (ERT or chaperonic) and other therapies, kidney disease biomarkers, and the levels of lyso-Gb3.

## 4. Discussion

In this single-center cohort of adult subjects with a clinical and a biochemical diagnosis of Fabry disease (FD) who were clinically characterized as having the classic and late-onset forms, we found an unbalanced relationship between the levels of the membrane-bound and of the soluble forms of IL-6 receptors. This study took advantage of the use of urinary exosomes as a tool to investigate ex vivo the levels of membrane-bound IL-6R, distinguishing it from the soluble form (sIL-6R) that circulates in the blood. Urinary exosomes are known to represent a useful tool to investigate markers of renal damage as they can derive from different segments of the nephrons. However, we herein used them as a systemic source of the membrane bound-IL-6R levels and not as markers of kidney status. This was conceived after the preliminary demonstration that the levels of the membrane-bound IL-6R in exosomes deriving from plasma and from urine were the same.

These results suggest a role of IL-6 in triggering the inflammatory status in FD. In fact, as the sIL-6R levels correlate with the pathogenic effect of IL-6 [20,21,22], the finding that, in FD patients, the levels of sIL-6Rs are consistently higher, as compared to healthy controls, points to its increased activation as a potential mediator of cell and tissue damage in FD. Moreover, the levels of sIL-6Rs were increased in subjects presenting with a classical form of FD (Figure 2). This evidence suggests that sIL-6Rs might correlate with the severity of the disease, as the classical form is featured by major clinical manifestations, which are rarely seen in the late-onset form [2]. However, the apparent lack of any effect of the therapy on sIL-6R levels deserves further investigations on the possible mechanisms that modulate the synthesis of this receptor.

Of note, as the homeostatic and protective action of IL-6 is mediated by the activation of the membrane receptor IL-6R in target cells, the slightly lower levels of it in FD patients further corroborate a scenario where IL-6 pathogenic signals prevail over its homeostatic action.

In this putative scenario, the activation of IL-6 soluble receptors, able to spread inflammatory signals all over the body, might in part explain some of the clinical presentations of FD, which feature multisystemic damage, also involving tissues not directly affected by Gb3 accumulation, as, for instance, the gastrointestinal system [23,24] or the bone [25,26]. Our data provide further evidence that other biological processes, besides the cellular alterations associated with Gb3 deposition, might participate in the irreversible tissue and organ damage featured in FD, similar to what was found, for instance, in the development of kidney fibrosis in FD [7].

To some surprise, we did not detect any increase in the circulating IL-6 concentrations, which were below the cut-off for normality in our clinical center (>7 pg/mL). The result was consistent in all patients, regardless of the *GLA* mutation status, age, gender, therapy, and the presence of a classical or a late form of FD.

Thus, in our cohort of FD adult patients, there was no over-production of IL-6, which is commonly observed in inflammatory and autoimmune states, where the cytokine increases several thousand-fold [27], but, rather, a physiological synthesis as detected in healthy individuals [28]. This result, in our view, further emphasizes the finding that, in FD patients, IL-6 soluble receptors are increased, suggesting a lack of balance in IL-6 homeostasis due to an alteration of IL-6 trans-signaling rather than to high levels of the cytokine, as seen in other pathological conditions [20,21,22]. The reasons for this alteration might be multiple, as the production of sIL-6Rs is finely regulated by different molecular mechanisms, including alternative mRNA splicing [15] and regulation of ADAM10 and ADAM17 proteases [16,17].

The lack of an overproduction of IL-6 in this group of FD subjects is only partially in contrast with what has been reported so far. In fact, in studies showing proteomic data from plasma of FD patients, IL-6 was not over-represented in the samples as compared to controls [29,30]. As regards other studies [10,11,12], they mainly considered the effects of ERT on IL-6 and looked for differences in the ERT-treated and not-treated subjects, without considering if IL-6 levels were above their normality cut-off.

In conclusion, this study provides novel information on alterations in the component of the IL-6 signaling that mediates the inflammatory action of the cytokine in patients with FD. Further specifically designed studies will be necessary to measure if the IL-6 signaling system is modified during the natural history of the disease. However, identifying a new potential biomarker of the inflammatory status of FD patients may be helpful in the patient’s follow up and also in the selection of already available therapeutic approaches able to control IL-6 trans-signaling [14].

## Figures and Tables

**Table 1:** Characteristics of the Fabry disease patients (n = 20) included in the study.

	Fabry Disease Patients (n = 20)
**Age** (years)	43 ± 16
**Sex**	60% females; 40% males
***GLA* mutations** (n of subjects):	
N215S	7
W287X	4
A73V	3
M290T	1
R301X	1
G35R	1
G212Pfs*18	1
Q279K	1
G360Wfs*15	1
**Disease form** (%)	30% Classical, 70% Late-onset
**Therapy** (%)	50% ERT, 20% Chaperonic, 30% none
**e-GFR CKD EPI** (mL/min’/1.73 mq)	87.0 ± 27.6
**Proteinuria** (>0.15 g/24 h)	45% yes, 55% no

ERT: enzyme replacement therapy; e-GFR CKD EPI: Estimated Glomerular Filtration Rate Chronic Kidney Disease Epidemiology Collaboration.

## Data Availability

Data are contained within the article and Supplementary Materials.

## Supplementary Materials

The following supporting information can be downloaded at: https://www.mdpi.com/article/10.3390/jcm13010218/s1, Figure S1: Flow cytometric analysis on urinary exosomes; Table S1: Flow cytometric analysis on urinary exosomes.
