# Supplementary material for: Increased Soluble Interleukin 6 Receptors in Fabry Disease
Source: J Clin Med. 2023 Dec 29;13(1):218. doi: 10.3390/jcm13010218 (PMC10780051; doi:10.3390/jcm13010218)

Supplementary Material

Table S1. Flow cytometric analysis on urinary exosomes performed using the CytoFLEX S flow cytometer (Beckman Coulter, USA). The following antibodies for exosomes analysis were used: monoclonal CD-63, FITC-conjugated (# 353006, BioLegend, Prodotti Gianni Srl) and rabbit monoclonal IL-6 receptor (ab222101, Abcam, Prodotti Gianni Srl) The primary antibody against IL-6R was detected by incubation with anti-rabbit IgG Alexa Fluor 647 conjugated (Thermo Fisher Scientific).

Subject Code	n° events IL6/uL	n° events CD63 /uL	n° events CD63+IL6/uL	Tot negative events /uL	Tot n° positive events /uL
**Fabry Patients**					
**F1**	516,12	54,74	10,43	75463,34	581,29
**F2**	438,67	58,68	2,79	55878,73	500,14
**F3**	966,82	93,22	26,63	84150,85	1086,68
**F4**	1830,61	35,69	15,3	80294,24	1881,6
**F5**	1145,33	73,89	13,2	79017,21	1232,42
**F6**	501,32	47,08	13,85	56136,26	562,24
**F7**	1988,28	89,45	46	79418,66	2123,74
**F8**	618,39	58,64	5,33	72772,98	682,36
**F9**	133,93	65,65	7,88	85584,08	1407,45
**F10**	2288,78	63,87	5,32	81150,44	2357,97
**F11**	1788,48	71,65	0	84031,92	1860,12
**F12**	1060,19	54,97	20,94	75700,25	1136,1
**F13**	1191,34	65,88	10,98	87596,19	1268,19
**F14**	1600,89	86,39	48,59	86312,81	1735,87
**F15**	587,89	44,54	1781	47289,18	650,23
**Control subjects**					
**C1**	6735,51	15,2	15,2	72264,26	6765,91
**C2**	5978,24	2,47		75030,23	5980,71
**C3**	6081,08	8,31	2,77	78691,32	6092,16
**C4**	2948,35	2,83	0	41282,54	2951,19
**C5**	3398,23	11,37	0	80801,18	3409,62
**C6**	7009,47	11,44	14,3	87545,4	7035,2
**C7**	4883,17	5,37	13,42	63710,8	4904,97
**C8**	6108,17	13,99	0	84173,29	6123,03
**C9**	8381,54	5,13	0	74840,84	8386,67
**C10**	1265,37	109,32	30,06	87635,97	1404,76
**C11**	859,85	61,95	4,96	77069,7	926,75
**C12**	1153,71	94,79	10,83	82522,83	1259,33
**C13**	1118,67	51,71	21,77	86418,05	1192,17
**C14**	527,11	46,28	18	59722,3	591,39
**C15**	517,73	18,03	10,3	62845,67	546,06

Figure S1. Flow cytometric analysis on urinary exosomes performed using the CytoFLEX S flow cytometer (Beckman Coulter, USA). Flow Cytometry calibration for exosomes detection was performed with fluorescent polystyrene beads Gigamix a mix 1:1 of Megamix FSC & SSC Plus of known sizes of 0.1, 0.16, 0.2, 0.24, 0.3, 0.5, and 0.9 µm (BioCytex, Marseille, France) through the "Violet side Scatter" (VSSC) and fluorescent FL1 channels. VSSC at 405 nm is used to discriminate background noise. The working gate was set between 50 and 150 nm, defined as the Exosome gate. The following antibodies for exosomes analysis were used: mouse monoclonal CD-81, PE-conjugated (#349506, BioLegend Prodotti Gianni Srl), mouse monoclonal CD-63, FITC-conjugated (# 353006, BioLegend, Prodotti Gianni Srl) and rabbit monoclonal IL-6 receptor (ab222101, Abcam, Prodotti Gianni Srl). The primary antibody against IL- 6R was detected by incubation with anti-rabbit IgG Alexa Fluor 647 conjugated (Thermo Fisher Scientific). Panel A: negative control; panel B: urine from a healthy subject; panel C: urine from a Fabry patient

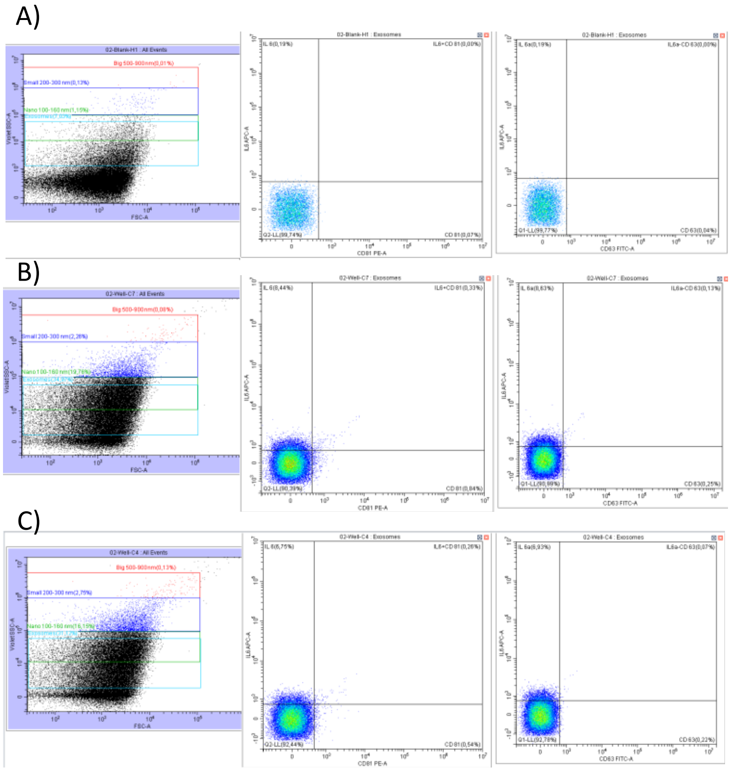

Supplement: Supplementary file 1 [file jcm-13-00218-s001.zip › jcm-2725101-supplementary.pdf]
